# Flourishing through Social Development Activities and Social Support: A Holistic Strategy for Problematic Smartphone Use

**DOI:** 10.1007/s11126-025-10126-w

**Published:** 2025-03-03

**Authors:** Orhan Koçak, Orhan Çevik, Orçun Muhammet Şimşek

**Affiliations:** 1 Department of Social Work, Faculty of Health Sciences, Istanbul University-Cerrahpasa, 34500 Istanbul, Türkiye; 2 Department of Social Work, Institute of Graduate Studies, Istanbul University-Cerrahpasa, 34320 Istanbul, Türkiye

**Keywords:** Social development activities, Perceived social support, Problematic smartphone use, General wellbeing, Flourishing

## Abstract

While some research has indicated the relationship between participation in social development activities (PSDA) and perceived social support (PSS) with problematic smartphone use (PSU), there has been a lack of studies examining these relationships through the lens of the flourishing theory, particularly among adolescents. To address this gap in the literature, this study conducted between April and May 2022 included 4965 high school students from 20 different cities in Türkiye. Researchers utilized questionnaires assessing participants' demographic information, levels of participation in social development activities, The Smartphone Application-Based Addiction Scale, The Short Version of the Scales of General Well-Being (GWB), and The Multidimensional Scale of Perceived Social Support as instruments. Structural Equation Modeling was employed for analyzing the relationships between variables using IBM SPSS v26 and AMOS v24 software packages. Hierarchical regression analysis was used to understand how GWB and PSS were associated with the relationships between PSDA and PSU. Findings indicated a predominantly negative relationship between PSDA and PSU, with GWB mediating this relationship negatively across all PSDA and PSS playing a contributory role in many indirect relationships between PSDA and PSU, if not directly. The importance of the approach in interventions aimed at addressing PSU was emphasized.

## Introduction

The first smartphone to feature a large touchscreen, full internet capabilities, sleek design, lightweight construction, and expandable memory card options was introduced by Steve Jobs 17 years ago [1]. Since then, smartphones, which have found their place in almost every household, are now nearly ubiquitous and can be found in almost every pocket. According to a report by Statista, smartphone subscribers increased from 3.7 billion to 6.3 billion between 2016 and 2021 [2]. While China leads in smartphone usage with 953.5 million users, Türkiye ranks 13th with 55.1 million users [3]. In terms of smartphone usage per capita, the United States ranks first at 82.2%, while Türkiye ranks 11th at 64.8% [4]. In Türkiye, 97.5% of internet users aged 16–64 own a smartphone, and internet access, which averages 4.5 h per day, is done via smartphones 95.5% of the time [5]. Furthermore, 94.7% of internet users access the internet via smartphones, with 80.7% using it for indirect information acquisition purposes. Additionally, 79.6% of video game players prefer using smartphones for gaming [5]. According to a report prepared by the Turkish Statistical Institute regarding technology usage among youth, the smartphone usage rate among early adolescents (ages 11–15) in Türkiye is 75% [6]. Of these, 88.8% use smartphones every day, while 9.8% use them at least once a week. However, adolescents primarily use smartphones for studying (81.5%) and participating in online classes (79.2%), while they use them least for social media (56.8%) and gaming purposes (64.4%). On weekdays, the average smartphone usage time is approximately 5 h, while on weekends, it reaches approximately 4 h. Furthermore, 43.5% of individuals check their phones at least once every 30 min, checking smartphones before sleep is the last activity for 42%, and for 36.4%, it is the first activity after waking up. Moreover, 30.3% continue to use their smartphones while watching television, 13.3% while eating, and 64.5% engage in at least one of these activities, with 5.2% engaging in all of them simultaneously. The age at which individuals begin using smartphones decreased from 10 in 2013 to 9 in 2021.

Smartphones possess numerous features, which enhance the quality of human life However, alongside the advantageous nature of smartphone usage, there are also disadvantages. Problematic smartphone use is defined as excessive or compulsive smartphone engagement that interferes with daily functioning, leading to negative psychological, social, or academic consequences, and it stands out as one of these disadvantages [7, 8]. A meta-analysis study conducted across 81 studies from 24 countries published between 2014 and 2020 found a global average score of 28.78 (min. 10 and max. 60, sd 4.16) for the level of problematic smartphone usage [9]. Cross-cultural studies have further highlighted significant variations in problematic smartphone usage, showing that cultural factors influence both the prevalence and patterns of smartphone use [10, 11]. These findings underscore the need for culturally sensitive interventions and policies. In Türkiye, this average was around 22 in 2014 and around 30 in 2020. According to another meta-analysis study conducted on 82 research papers published between 2005 and 2021, the prevalence of problematic smartphone usage was 26% between 2015 and 2019, increasing to 34.5% between 2020 and 2021. The overall prevalence rate between 2005 and 2021 is 27%. Furthermore, a meta-analysis of 26 studies found that the prevalence of problematic smartphone usage among adolescents is approximately 22% [12].

Family, friends, and receiving approval from people in the immediate social circle are considered fundamental needs for every individual. Failure to meet these needs in healthy ways leads to a disruption in well-being and the emergence of physical and mental disorders [13]. With the onset of the Covid-19 pandemic, the fulfillment of these needs has been restricted, and it has been observed globally, particularly an increase in mental disorders related to the misuse of technology [14]. Studies have shown that rapidly increasing problematic smartphone use is one of these disorders [15]. Therefore, it can be said that preventing problematic smartphone use contributes to maintaining general well-being, and maintaining general well-being is dependent on meeting psychological basic needs. In this context, this study aims to examine the effects of participation in social development activities, which are fundamental needs, and family and friend social support on general well-being and problematic smartphone use in adolescents, a disadvantaged group.

### Problematic Smartphone Use

Although not yet included in DSM-5 or ICD-11, problematic smartphone use (PSU) is defined by some researchers as the inability to regulate the time spent on smartphones, which is associated with adverse effects on daily life, particularly in the long term [16]. Over the past twenty years, this phenomenon has begun to manifest itself in the literature under various names. Especially in the existing literature, this situation has been described with concepts such as problematic smartphone use and dependence, mobile phone addiction, smartphone addiction, problematic mobile phone usage, impulsive cell phone use, and excessive mobile phone use [17–27]. Although the negative effects of smartphones are described with different concepts, there is not enough evidence to consider smartphones as addiction [8, 28]. Moreover, as a relatively newer and different concept, the concept of problematic smartphone use and dependence (PSUD) is also found in the literature [29]. While each concept makes a significant contribution to the literature, the concept of problematic smartphone use was preferred in this study in order to organize this conceptual breadth in the literature.

### General Wellbeing

One of the key concepts associated with problematic smartphone use is general wellbeing [30]. General wellbeing (GWB) is defined as an overall assessment of life quality reflecting a high-level psychological experience, social function, and adaptation status of the individual. This concept also draws attention to the socialization of the individual beyond the subjective and psychological aspects of wellbeing. Longo et al. (2018) worked on a reference scale aiming to define general wellbeing [31]. The researchers stated that general wellbeing was composed of 14 separate variables according to the literature they obtained from previous studies. Happiness, vitality, calmness, optimism, engagement, mindfulness, acceptance, self-worth, competence, growth, purpose, significance, adaptation, and connection were variables defining general well-being. Each variable is interrelated and contributes collectively to the overarching concept of general well-being. Then, these factors constituting general wellbeing were tested on the Turkish sample, and it was observed that except for the calmness factor, the other 13 factors were found to be related to general wellbeing [32]. In conclusion, general wellbeing is a profound psychological experience representing a comprehensive evaluation of an individual's quality of life; this evaluation includes social functionality and adaptation status [33].

### Participation in Social Development Activities

Participation in social development activities is another key factor associated with reducing problematic smartphone use [34]. Social skills form the foundation of success in real-life situations [35]. The development of social skills often requires specific and intentional motivation, distinct from the general motivation for participation in activities, as it involves conscious effort and practice to enhance interpersonal interactions [36]. Participation in activities is considered a key component of social development, as it plays a significant role in building social skills and facilitating the socialization process [37]. Participation in activities supports social engagement and particularly opens up space for new meaningful experiences [37]. These new meaningful experiences encompass a wide range of social activity repertoire, from making new friends to participating in recreational activities [38]. When addressing participation in social development activities (PSDA), it is important to consider a holistic structure with its components. By holistic structure, we mean the necessity of emphasizing the contexts that constitute the activities. This is because the social development activities addressed in the current study represent a holistic structure together with seven different activities. Although religious, volunteer, physical, social, cultural, educational, and e-sports activities may appear as different activities, each can be considered as supportive elements of social development. Strengthening social ties among individuals gathered around beliefs is facilitated by religious activities [39]. Increasing social interactions by promoting social responsibilities is supported through volunteer activities [40]. Enhancing social cohesion through exercising together and thereby accelerating social integration is associated with physical activities [41]. Improving communication skills, fostering empathy, and enabling collaboration through coming together can be linked to the efficacy of social activities [38]. Acquiring knowledge about different cultures and fostering tolerance is associated with cultural activities [42]. Participation in educational activities involves engaging in opportunities designed to enhance knowledge acquisition and skill development, which may support both personal and academic growth [43]. Lastly, e-sports activities are associated with the development of teamwork and strategic thinking and problem-solving skills [44]. It is beneficial to note that these different activities focus on social development. As mentioned, social activities are activities based on mutual interactions among individuals. However, other activities are also included in social activities. Therefore, we believe that creating an umbrella concept is necessary. In this study, we used the concept of social development activities as an umbrella concept.

### Perceived Social Support

Perceived social support is another crucial factor associated with problematic smartphone use [45]. Social relationships are an indispensable part of human life. Particularly, supportive social relationships are considered potentially important for being healthy [46]. Social support is widely conceptualized as support perceived from close social circles in response to stressful events [45, 47]. In other words, it signifies individuals' subjective evaluations of the readiness of their close circles, such as friends and family, to provide support [48]. Both family and friends play a crucial role in an individual's personal and professional development throughout their life. Because receiving both family (FaSS) and friend (FrSS) social support are associated with better social well-being [49]. Research suggests that a supportive environment strengthens an individual's ability to cope with life's challenges and, therefore, is positively correlated with their overall well-being [50]. While some social support studies emphasize the importance of peer support against stressful family life, others show that peer support occupies less space in adolescents' lives than predicted [51, 52]. This refers to the multidimensional structure of social support and represents the potential to be influenced by different situations.

### Theoretical Background and The Present Research

The theory of flourishing has long been addressed as a significant subject within positive psychology [53]. Keyes has been a pioneer in defining and developing the concept of flourishing, characterizing it as positive emotions and psychosocial well-functioning in life [54]. The dynamics of flourishing have been particularly shaped around concepts such as positive emotions, meaningful activities, strong relationships, meaning, and accomplishment in the literature [55]. These concepts have aided in linking flourishing with healthy mental health. This is because the presence of mental health is described as flourishing, while the absence of mental health is referred to as languishing [56]. However, resolving mental illnesses does not necessarily imply the attainment of an enhanced life. In other words, overcoming problems does not rely solely on treatment but also necessitates promoting a balanced life in which individuals may experience happiness and personal growth [56].

In the context of this study, flourishing theory provides a framework to understand the interconnected roles of general well-being (GWB) as a mediator, participation in social development activities (PSDA), and perceived social support (PSS) as a covariate in addressing problematic smartphone use (PSU). Participation in social development activities aligns with the flourishing concepts of meaningful activities, positive emotions, and strong relationships, as it provides individuals with opportunities to engage in fulfilling experiences, strengthen social bonds, and derive a sense of accomplishment. General well-being acts as a core element of flourishing, and is associated with resilience and life satisfaction, which in turn may be linked to a decreased reliance on excessive smartphone use as a coping mechanism. Perceived social support complements these effects by fostering strong interpersonal relationships and lessening the likelihood of feelings of loneliness or isolation, factors that are closely tied to PSU.

The present study aims to investigate the mediating role of general well-being in the relationship between social developmental activities and problematic smartphone use, while also focusing on the association of family and friend social support, while acknowledging the theory of flourishing. In this context, we have formulated three research questions.

#### RQ-1: Is PSDA Associated with PSU?

Participation in social development activities is considered a supportive element of a fulfilling life [57], as engagement in such activities is associated with strengthening positive emotions and meaning [58]. Consequently, individuals may cope more effectively with challenges they encounter [59]. Therefore, encouraging participation in social development activities could be linked to problematic smartphone use. Previous literature has examined the associations between participation in social development activities and problematic smartphone use [60–66].

#### RQ-2: Is GWB a Mediator in the Relationship between PSDA and PSU?

Many researchers in the field of positive psychology acknowledge that flourishing encompasses the evolving state of well-being [55, 67–70]. While taking numerous steps to support well-being is associated with a flourishing life, it may also be linked to addressing many problems simultaneously [33, 63]. Previous literature has addressed participation in activities due to its association with well-being [71–79]. Participation in activities is akin to the leading elements of an evolving life and is an indispensable part of general well-being. Promoting general well-being may be associated with addressing behavioral problems such as problematic smartphone use [30, 63, 80, 81]. This relational association suggests that general well-being may serve as a mediator between social developmental activities and problematic smartphone use.

#### RQ-3: Is PSS a Covariate in the Relationship between PSDA and PSU?

**Fig. 1:**
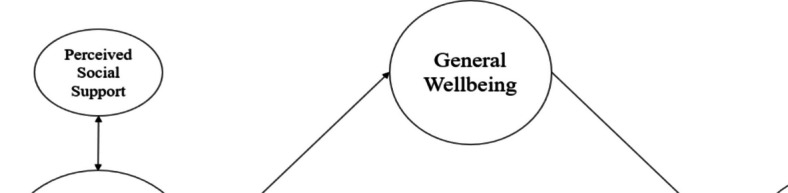
Research model

Research examining concepts associated with achieving a flourishing had relatively less evidence regarding the role of social support [68]. However, a series of studies presented findings indicating an association between perceived social support and well-being [82–86]. Flourishing is vital for individual development, but is individual development alone sufficient for flourishing? In other words, enhancing abilities to overcome problems may contribute to positive outcomes, but lacking a supportive environment might lessen this association. Particularly, environmental factors such as family and friends serve as supportive elements for a healthy life and are associated with overcoming problems [68, 87]. Previous research has suggested that social support is linked to development, supports well-being, and is associated with buffering behavioral problems such as problematic smartphone use [49, 88–91]. In this context, it is believed that social support mechanisms such as family and friend social support may play a role in shaping the associations observed in the current study Fig. 1.

## Materials and Methods

### Study Design and Participants

This study was conducted using the convenience sampling technique and a correlational research design [92]. A questionnaire was delivered to a sample of 4965 high school students from 20 different cities in Türkiye in April and May 2022. The participants' mean age was 15.87 (SD. 1.27) and the questionnaire was administered using a web-based survey. The necessary permissions for the research were obtained from the Ministry of Youth and Sports and the Ministry of National Education. The study was reviewed and approved by the Ethics Committee of Istanbul Nisantasi University with the protocol code 2022/13, and the approval date was 25 March 2022. The sample size in this study is representative of the approximately 6.5 million high school students in Türkiye, with a margin of error of 1.83% and a confidence interval of 95% according to the Sample Size Calculator [93].

### Measures

In this study, a questionnaire consisting of items scaling participation level in social development activities within demographic questionnaire prepared by researchers, The Smartphone Application-Based Addiction Scale, The Short Version of the Scales of General Well-Being and The Multidimensional Scale of Perceived Social Support were used as data collection tools.

#### Participation in Social Development Activities Questionnaire

This is not a scale that has undergone validity and reliability testing. It was prepared by researchers in a similar format with a scale to understand the frequency of participation in social development activities. There are a total of 7 questions regarding the frequency of participation in Social, Physical, Cultural, Educational, Voluntary, Religious, and E-Sport activities on a 5-point Likert scale (1: Never and 5: Always), and each question is analyzed independently. The items include: "I participate in social activities (spending time with friends, meeting for conversations, visiting someone's home, talking on the phone with relatives/friends, etc.)", "I participate in physical activities (exercising, fitness, swimming, dancing, walking, biking, etc.)", "I participate in cultural activities (watching TV/movies/series, going to the cinema, going to the theater, listening to music, etc.)", "I participate in educational activities (taking extracurricular courses, reading books, participating in online courses, etc.)", "I participate in voluntary activities (planting trees, visiting nursing homes, supporting those in need, taking care of stray animals, etc.)", "I participate in religious activities (engaging in collective worship, reading religious texts, following religious discussions, etc.)", and "I participate in e-sport activities (playing group games via computer/tablet/smartphone)".

#### The Smartphone Application-Based Addiction Scale (SABAS)

This is an assessment tool developed by Csibi et al. in 2018 [94]. It aims to evaluate the potential addiction risk associated with smartphone applications. Gökler & Bulut conducted a study in 2019 to establish the scale's validity and reliability in the Turkish context [95]. The scale comprises six questions, with responses given on a 6-point Likert scale ranging from strongly disagree (1) to strongly agree (6). Example statements in the scale include “My smartphone is the most important thing in my life.” and “I have conflicts with my family (or friends) due to my smartphone use.” In our study, the scale was found to be reliable (Cronbach's alpha = 0.78)**.**

#### The Short Version of the Scales of General Well-Being (SVSGW)

Validity and reliability for Turkish version of this scale, developed by Longo, Coyne, and Joseph (2018), were conducted by Kalafatoğlu and Balcı Çelik (2020) [31, 32]. It is a five-point Likert scale consisting of one factor and 13 items (1-Strongly Disagree to 5-Strongly Agree). The lowest possible score on the scale is 5, while the highest is 65. A high score on the scale indicates a high level of general well-being. The scale items are as "I feel happy.", "I feel that I am developing.", "I have a purpose.". In our study, the scale was found to be reliable (Cronbach's alpha = 0.88).

#### The Multidimensional Scale of Perceived Social Support (MSPSS)

4-item Family Social Support and 4-item Friend Social Support dimensions of this 12-item and 7-point likert-type scale were used in the study by excluding 4-item Special Friend Social Support dimension. The Turkish validity and reliability of the scale developed by Zimet et al. (1988) was done by Eker et al. (2001) [96, 97]. Both dimensions are normally distributed and reliable (Cronbach's alpha = 0.88 for Family Social Support and 0.90 for Friend Social Support) in this study.

### Data Analysis

The SPSS v26 program was used for descriptive statistics, bivariate correlation, and reliability analyses in the study. For normality, validity, and structural equation modeling analyses, the SPSS Amos v24 program was utilized. Structural Equation Modeling (SEM) is used to examine the relationships between multiple variables simultaneously, providing insights into direct and indirect effects [98–100]. The analyses revealed that the scales are reliable, and the dataset follows a normal distribution. The 13th item of General Wellbeing Scale ("I feel close and connected to the people around me.") had a low factor loading in outputs of confirmatory factor analysis and was therefore not included in the model. To better understand relationships among variables, a hierarchical regression method was employed within the structural equation model in the study. Hierarchical regression analysis is a statistical method to understand the contribution of different variables by adding them into the model step by step [101, 102]. In all steps, it was observed that the goodness of fit values of the models, except for cmin/df, were within acceptable ranges. Due to the sensitivity of the cmin/df value to sample size and the acceptability of other values within the range, the magnitude of this value has not been taken into account [103]. The 2000-sample bootstrap method based on 95% bias-corrected confidence intervals was employed for examining associations involving indirect effects.

## Results

### Descriptive Statistics

Table 1 presented information regarding the sociodemographic characteristics of the participants and scale averages. It was observed that the majority of the participants, with an average age of 15.87 and were female (56.9%). When examining the continuous variables, the participation level in social (3.51), cultural (3.20), and physical (3.49) activities is above the scale average. It was understood that the highest participation was in social activities (3.51), while the lowest participation was in voluntary activities (2.25). It was found that the level of family and friend social support was above the scale average (4.0) and that friend social support (5.17) was higher than family social support (5.09). The average level of general well-being (3.31) was above the scale average (3.0). The level of problematic smartphone use (3.0) appeared to be the same as the scale average (3.0). Skewness and kurtosis values indicated that the dataset was normally distributed.

**Table 1:** Descriptive statistics

Variables		f	%	Mean	SD	Skew	Kurt
**Age**	13–20			15.87	1.27	0.28	−1.92
**Gender**	Female Male	2827 2138	56.9 43.1			0.19	−0.63
**Social Development Activities**							
Social Activities Cultural Activities Physical Activities Educational Activities Voluntary Activities Religious Activities E-Sport Activities	1–5			3.51 3.20 3.49 2.88 2.25 2.78 2.33	1.07 1.27 1.14 1.21 1.18 1.27 1.35	−0.33 −0.05 −0.28 0.15 0.77 0.20 0.63	−0.51 −1.04 −0.79 −0.88 −0.21 −0.94 −0.83
**Perceived Social Support**							
Family Social Support Friend Social Support	1–7			5.09 5.17	1.56 1.53	−1.04 −0.70	0.33 −0.36
**General Wellbeing**	1–5			3.31	0.07	−0.34	−0.15
**Problematic Smartphone Use**	1–5			3.00	0.93	0.03	−0.65
**Total**		4965	100				

### Correlation

Table 2 displayed the relationships between the variables in the study. According to the findings, family social support had a positive significant relationship with all PSDA (r = 0.24, 0.21, 0.18, 0.19, 0.17, 0.20, and 0.03, *p* < 0.05), friend social support (r = 0.32, *p* < 0.05), and GWB (r = 0.59, *p* > 0.05), while having a negative significant relationship with PSU (r = −0.26, *p* < 0.05). Similarly, friend social support had a positive significant relationship with all PSDA (r = 0.38, 0.23, 0.21, 0.11, 0.09, 0.03, and 0.07, *p* < 0.05) and GWB (r = 0.32, *p* < 0.05), but had no significant relationship with PSU (r = −0.01, *p* > 0.05). A negative significant relationship was found between GWB and PSU (r = −0.40, *p* < 0.05), and all PSDA except for social and cultural activities (r = 0.02 and 0.00, *p* > 0.05), were associated with PSU (r = −0.11, −0.14, −0.07, −0.13, and 0.08, *p* < 0.05).

**Table 2:** Correlations

Variables	1	2	3	4	5	6	7	8	9	10
**1.** Social A **2.** Cultural A **3.** Physical A **4.** Educational A **5.** Voluntary A **6.** Religious A **7.** E-sport A **8.** FaSS **9.** FrSS **10.** GWB **11.** PSU	1 0.37* 0.34* 0.19* 0.19* 0.11* 0.11* 0.24* 0.38* 0.28* 0.02	1 0.38* 0.24* 0.31* 0.16* 0.21* 0.21* 0.23* 0.41* 0.00	1 0.33* 0.28* 0.10* 0.13* 0.18* 0.21* 0.25* −0.11*	1 0.34* 0.21* 0.03* 0.19* 0.11* 0.31* −0.14*	1 0.29* 0.17* 0.17* 0.09* 0.30* −0.07*	1 0.15* 0.20* 0.03* 0.32* −0.13*	1 0.03* 0.07* 0.10* 0.08*	1 0.32* 0.59* −0.26*	1 0.32* −0.01	1 −0.40*

**Notes.** *significant at 0.05 level. *PSU* Problematic Smartphone Use *GWB* General Wellbeing, *FrSS* Friend Social Support, *FaSS* Family Social Support, *A* Activities

### SEM

#### Step 1: Social Development Activities

**Fig. 2:**
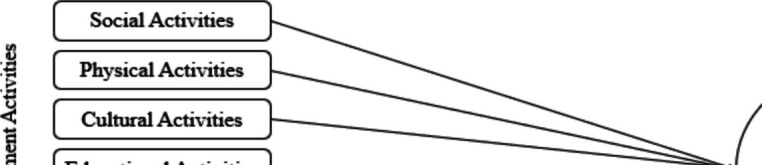
Step 1 model

In Fig. 2, the diagram of the first step of the research model and the results of the path analysis were presented in Table 3. When examining the findings, it was observed that among PSDA, the activities that showed the strongest negative associations with PSU were, in order, physical (β = −0.13, %95 CI [−0.16,−0.10]), religious (β = −0.11, %95 CI [−0.14,−0.08]), and educational activities (β = −0.10, %95 CI [−0.13,−0.07]). However, it was found that among the activities examined, e-sport activities had the strongest positive association with problematic smartphone use (β = 0.11, 95% CI [0.08, 0.14]), followed by social activities (β = 0.06, 95% CI [0.03, 0.09]) and cultural activities (β = 0.07, 95% CI [0.04, 0.10]). Volunteer activities did not have a significant negative association with PSU. Estimation value of squared multiple correlation (R^2^) was 0.05 for PSU in this model.

**Table 3:** Step 1 outputs

Variables	Model		Effect	LB	UB	SE	*β*	p
Social A Physical A Cultural A Educational A Voluntary A Religious A E-sport A	→	Problematic Smartphone Use	Total	0.03 −0.16 0.04 −0.13 −0.04 −0.14 0.08	0.09 −0.10 0.10 −0.07 0.01 −0.08 0.14	0.01 0.01 0.01 0.01 0.01 0.01 0.01	0.06 −0.13 0.07 −0.10 −0.01 −0.11 0.11	0.001* 0.001* 0.001* 0.001* 0.528 0.001* 0.001*

#### Step 2: General Wellbeing

**Fig. 3:**
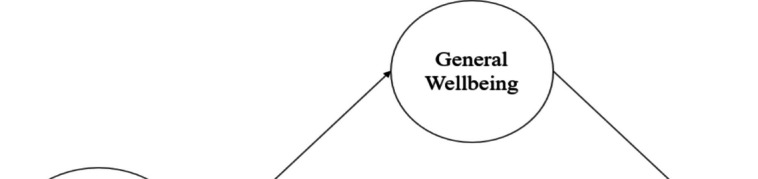
Step 2 model

Figure 3 illustrated the schematic representation of the second phase of the research model, while Table 4 outlined the outcomes of the path analysis. Within this framework, wherein general well-being serves as a mediating factor, it was evident that GWB acted as a mediator for all relationships between PSDA and PSU, except for cultural and e-sports activities. Notably, the positive direct association of social (β = 0.10, 95% CI [0.07, 0.13]) and volunteer (β = 0.01, 95% CI [−0.01, 0.04]) activities with PSU was found to have a negative indirect association via GWB (β = −0.03, 95% CI [−0.04,.−02]; β = −0.02, 95% CI [−0.03, −0.01], respectively). Conversely, the positive direct association of e-sports activities with PSU (β = 0.10, 95% CI [0.07, 0.13]) was found to have an insignificant indirect association through GWB (β = 0.00, 95% CI [−0.00, 0.01]). Moreover, the negative direct associations of physical (β = −0.03, 95% CI [−0.06, 0.00]) and religious (β = −0.03, 95% CI [−0.06, 0.00]) activities with PSU were amplified through GWB, manifesting as more robust negative indirect associations (β = −0.09, 95% CI [−0.10, −0.08]; β = −0.07, 95% CI [−0.08, 0.06], respectively). Interestingly, the negative direct association of educational activities with PSU (β = −0.05, 95% CI [−0.08,.−02]) was found to have a weaker negative indirect association facilitated by GWB (β = −0.04, 95% CI [−0.06,.−03]). Upon examining both direct and indirect associations, it became evident that GWB's mediation played a pivotal role in the overall associations of physical (β = −0.13, 95% CI [−0.16,.−09]), volunteer (β = −0.01, 95% CI [−0.04, 0.02]), and religious (β = −0.10, 95% CI [−0.14,.−07]) activities with PSU. R^2^ was 0.15 for PSU and 0.25 for GWB in this model. Consequently, the findings support the hypothesis that GWB mediated the relationships between PSDA and PSU, as proposed in the second research question.

**Table 4:** Step 2 outputs

Variable		Model			Effect	LB	UB	SE	*β*	*p*
Social A Physical A Cultural A Educational A Voluntary A Religious A E-sport A		→		Problematic Smartphone Use	Total	0.03 −0.16 0.03 −0.14 −0.04 −0.14 0.07	0.10 −0.09 0.10 −0.07 0.02 −0.07 0.14	0.01 0.01 0.01 0.01 0.01 0.01 0.01	0.06 −0.13 0.06 −0.10 −0.01 −0.10 0.10	0.001* 0.001* 0.001* 0.001* 0.560 0.001* 0.001*
Social A Physical A Cultural A Educational A Voluntary A Religious A E-sport A		→		Problematic Smartphone Use	Direct	0.07 −0.06 0.04 −0.08 −0.01 −0.06 0.07	0.13 −0.00 0.10 −0.02 0.04 −0.00 0.13	0.01 0.01 0.01 0.01 0.01 0.01 0.01	0.10 −0.03 0.07 −0.05 0.01 −0.03 0.10	0.001* 0.046* 0.001* 0.001* 0.389 0.033* 0.001*
Social A Physical A Cultural A Educational A Voluntary A Religious A E-sport A	→	General Wellbeing	→	Problematic Smartphone Use	Indirect	−0.04 −0.10 −0.01 −0.06 −0.03 −0.08 −0.00	−0.02 −0.08 0.00 −0.03 −0.01 −0.06 0.01	0.00 0.00 0.00 0.00 0.00 0.00 0.00	−0.03 −0.09 −0.00 −0.04 −0.02 −0.07 0.00	0.001* 0.001* 0.275 0.001* 0.001* 0.001* 0.384

Notes. *LB&UB* Lower & Upper Bounds, *β* Standardized Estimates, *SE* Standard Error, *A* Activities

Model fit values. CMIN/DF: 9,69, RMSEA: 0.04, SRMR: 0.03, GFI: 0.96, AGFI: 0.95, NFI: 0.93, TLI: 0.93, CFI: 0.94

#### Step 3: Perceived Social Support

##### Level 1: Family Social Support

**Fig. 4:**
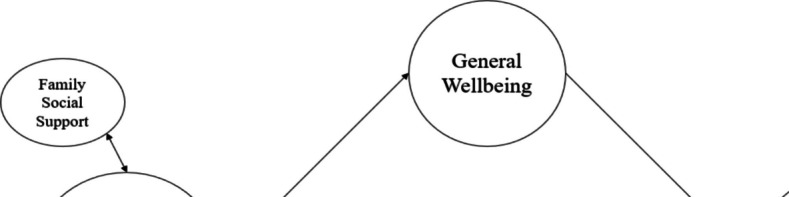
First level in third step (Family Support)

In Fig. 4, the schematic representation of the first level where the covariate role of family support (FaSS), a sub-dimension of perceived social support, was examined solely in the third stage of the research model, was presented, while Table 5 summarized the results of the path analysis. Within this framework where family social support served as a covariate and General Well-Being (GWB) serves as a mediating factor, it was found that the direct negative association of FaSS with PSU (β = −0.07, 95% CI [−0.12, −0.03]) was significant, and its indirect association via GWB was also negatively amplified (β = −0.13, 95% CI [−0.16, −0.03]). Compared to the previous step, the covariate role of FaSS on the direct association of social activities with PSU was positively strengthened (β = 0.11, 95% CI [0.07, 0.14]), and on the direct association of physical activities with PSU was negatively enhanced (β = −0.04, 95% CI [−0.07, −0.00]) by 0.1 degree. No change was observed for other PSDAs. When indirect associations were examined, it was observed that the negative indirect associations of all PSDAs with PSU, except for cultural and e-sport activities, weakened with the inclusion of FaSS in the model. A similar situation applies to total associations, except for voluntary and e-sport activities. The R^2^ value was 0.15 for PSU and 0.41 for GWB in this model. Therefore, the findings supported the hypothesis regarding the covariate role of perceived social support on the relationships between PSDAs and PSU, as demonstrated through the results for FaSS.

**Table 5:** Step 3 outputs (Family Support)

Variable		Model			Effect	LB	UB	SE	*β*	*p*
Social A Physical A Cultural A Educational A Voluntary A Religious A E-sport A FaSS		→		Problematic Smartphone Use	Total	0.06 −0.14 0.04 −0.12 −0.03 −0.11 0.06 −0.25	0.13 −0.07 0.11 −0.05 −0.03 −0.04 0.13 −0.18	0.01 0.01 0.01 0.01 0.01 0.01 0.01 0.01	0.09 −0.11 0.07 −0.08 −0.00 −0.07 0.10 −0.21	0.001* 0.001* 0.001* 0.001* 0.942 0.001* 0.001* 0.001*
Social A Physical A Cultural A Educational A Voluntary A Religious A E-sport A FaSS		→		Problematic Smartphone Use	Direct	0.07 −0.07 0.04 −0.09 −0.01 −0.06 0.07 −0.12	0.14 −0.00 0.11 −0.02 0.05 0.00 0.13 −0.03	0.01 0.01 0.01 0.01 0.01 0.01 0.01 0.02	0.11 −0.04 0.07 −0.05 0.01 −0.03 0.10 −0.07	0.001* 0.030* 0.001* 0.001* 0.385 0.050* 0.001* 0.001*
Social A Physical A Cultural A Educational A Voluntary A Religious A E-sport A FaSS	→	General Wellbeing	→	Problematic Smartphone Use	Indirect	−0.02 −0.08 −0.01 −0.04 −0.03 −0.05 −0.00 −0.16	−0.00 −0.05 0.01 −0.02 −0.00 −0.03 0.00 −0.03	0.00 0.00 0.00 0.00 0.00 0.00 0.00 0.00	−0.01 −0.07 0.00 −0.03 −0.01 −0.04 −0.00 −0.13	0.001* 0.004* 0.942 0.001* 0.001* 0.001* 0.896 0.001*

Notes. *LB&UB* Lower & Upper Bounds, *β*:Standardized Estimates, *SE* Standard Error, *A* Activities

Model fit values. CMIN/DF: 9.06, RMSEA: 0.04, SRMR: 0.03, GFI: 0.95, AGFI: 0.94, NFI: 0.94, TLI: 0.93, CFI: 0.94.

##### Level 2: Friend Social Support

**Fig. 5:**
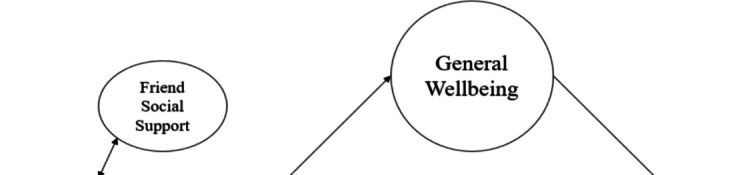
Second level in third step (Friend Social Support)

Figure 5 illustrated the schematic representation of the second level, where the covariate role of friend social support (FrSS), a sub-dimension of perceived social support, was examined in isolation during the third step of the research model. Meanwhile, Table 6 summarized the results of the path analysis. In this framework, where friend social support served as a covariate and a mediating factor for general well-being (GWB), it was found that the positive direct association of FrSS with PSU (β = 0.06, 95% CI [0.02, 0.09]) was significant, while its indirect association through GWB was again significant but negative (β = −0.07, 95% CI [−0.08, −0.05]). Comparing to the previous step, it was observed that there was a positive change in the direct association of social activities with PSU due to the covariate role of FrSS (β = 0.08, 95% CI [0.04, 0.12]). No change was observed for other PSDAs. When indirect associations were examined, it was seen that only the negative indirect associations of social activities with PSU weakened with the inclusion of FrSS in the model. A similar situation applied to the changes in positive total associations, primarily limited to social and cultural activities. R^2^ was 0.15 for PSU and 0.28 for GWB in this model. Therefore, the findings suggested that the hypothesis regarding the covariate role of perceived social support on the relationships between PSDAs and PSU via FrSS was not supported.

**Table 6:** Step 3 outputs

**Variable**		**Model**			**Effect**	**LB**	**UB**	**SE**	***β***	**p**
Social A Physical A Cultural A Educational A Voluntary A Religious A E-sport A FrSS		→		Problematic Smartphone Use	Total	0.03 −0.16 0.03 −0.14 −0.04 −0.14 0.07 −0.04	0.10 −0.09 0.10 −0.07 0.02 −0.07 0.14 0.02	0.01 0.01 0.01 0.01 0.01 0.01 0.01 0.01	0.07 −0.13 0.07 −0.10 −0.01 −0.10 0.10 −0.01	0.001* 0.001* 0.001* 0.001* 0.552 0.001* 0.001* 0.528
Social A Physical A Cultural A Educational A Voluntary A Religious A E-sport A FrSS		→		Problematic Smartphone Use	Direct	0.04 −0.07 0.03 −0.09 −0.01 −0.06 0.07 0.02	0.12 −0.00 0.10 −0.02 0.05 0.00 0.13 0.09	0.01 0.01 0.01 0.01 0.01 0.01 0.01 0.01	0.08 −0.03 0.07 −0.05 0.01 −0.03 0.10 0.06	0.001* 0.039* 0.001* 0.001* 0.323 0.049* 0.001* 0.002*
Social A Physical A Cultural A Educational A Voluntary A Religious A E-sport A FrSS	→	General Wellbeing	→	Problematic Smartphone Use	Indirect	−0.03 −0.10 −0.01 −0.06 −0.04 −0.08 −0.00 −0.08	−0.00 −0.07 0.01 −0.03 −0.01 −0.06 0.01 −0.05	0.00 0.00 0.00 0.00 0.00 0.00 0.00 0.00	−0.01 −0.09 −0.00 −0.04 −0.02 −0.07 0.00 −0.07	0.008* 0.001* 0.699 0.001* 0.001* 0.001* 0.299 0.001*

Notes. *LB&UB* Lower & Upper Bounds, *β *Standardized Estimates, *SE* Standard Error, *A* Activities

Model fit values. CMIN/DF: 8.47, RMSEA: 0.03, SRMR: 0.03, GFI: 0.96, AGFI: 0.95, NFI: 0.94, TLI: 0.94, CFI: 0.95.

##### Level 3: Family and Friend Social Support

**Fig. 6:**
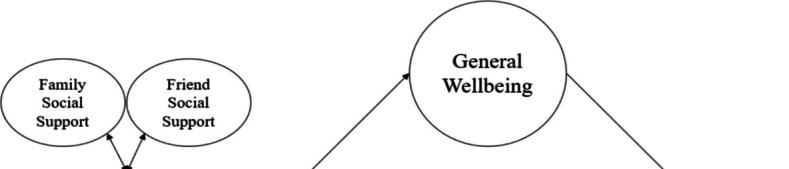
Third level in third step (Family and Friend Social Support)

Figure 6 provided a visual representation of the third tier, where both family social support (FaSS) and friend social support (FrSS), components of perceived social support, were collectively analyzed for their covariate effects during the third phase of the research model. Meanwhile, Table 7 offered a summary of the outcomes derived from the path analysis. Within this framework, where both family and friend social support functioned as covariates and mediators for general well-being (GWB), it was observed that the negative direct association of FaSS and PSU (β = −0.09, 95% CI [−0.13, −0.04]) was statistically significant, along with its negative indirect association through GWB (β = −0.13, 95% CI [−0.15, −0.11]). Conversely, FrSS exhibited a notable positive direct association with PSU (β = 0.07, 95% CI [0.03, 0.11]), accompanied by a similarly significant but negative indirect association via GWB (β = −0.03, 95% CI [−0.04, −0.02]). When contrasting with their direct associations in the prior step, there was a favorable change in the direct association of social activities with PSU within the covariate roles of FaSS and FrSS (β = 0.09, 95% CI [0.05, 0.12]), while an increase was observed in the direct negative association of physical activities with PSU (β = −0.04, 95% CI [−0.07, −0.00]). Furthermore, the previously substantial negative direct association of religious activities with PSU no longer had significance (β = −0.02, 95% CI [−0.06, 0.00]). No notable changes were detected for other social determinant activities (PSDAs). Upon examining indirect associations, there were no significant changes for cultural, voluntary, and e-sports activities. The formerly significant direct association of social activities with PSU lost significance. Moreover, there was a weakening in the degree of indirect associations of physical, educational, and religious activities. R2 was 0.15 for PSU and 0.42 for GWB within this model. Thus, the findings supported the hypothesis regarding the covariate role of perceived social support on the relationships between PSDAs and PSU.

**Table 7:** Step 3 outputs

Variable		Model			Effect	LB	UB	SE	*β*	*p*
Social A Physical A Cultural A Educational A Voluntary A Religious A E-sport A FaSS FrSS		→		Problematic Smartphone Use	Total	0.04 −0.15 0.04 −0.12 −0.03 −0.10 0.06 −0.26 0.00	0.12 −0.08 0.11 −0.05 0.03 −0.04 0.13 −0.18 0.08	0.01 0.01 0.01 0.01 0.01 0.01 0.01 0.01 0.01	0.08 −0.11 0.07 −0.08 −0.00 −0.07 0.10 −0.22 0.04	0.001* 0.001* 0.001* 0.001* 0.001* 0.976 0.001* 0.001* 0.029*
Social A Physical A Cultural A Educational A Voluntary A Religious A E-sport A FaSS FrSS		→		Problematic Smartphone Use	Direct	0.05 −0.07 0.39 −0.09 −0.01 −0.06 0.07 −0.13 0.03	0.12 −0.00 0.10 −0.02 0.05 0.00 0.13 −0.04 0.11	0.01 0.01 0.01 0.01 0.01 0.01 0.01 0.02 0.01	0.09 −0.04 0.07 −0.05 0.01 −0.02 0.10 −0.09 0.07	0.001* 0.021* 0.001* 0.001* 0.303 0.098 0.001* 0.001* 0.001*
Social A Physical A Cultural A Educational A Voluntary A Religious A E-sport A FaSS FrSS	→	General Wellbeing	→	Problematic Smartphone Use	Indirect	−0.01 −0.08 −0.00 −0.04 −0.03 −0.05 −0.00 −0.15 −0.04	0.00 −0.05 0.01 −0.02 −0.01 −0.03 0.00 −0.11 −0.02	0.00 0.00 0.00 0.00 0.00 0.00 0.00 0.01 0.00	−0.00 −0.07 0.00 −0.03 −0.02 −0.04 0.00 −0.13 −0.03	0.218 0.001* 0.678 0.001* 0.001* 0.001* 0.991 0.001* 0.001*

Notes. *LB&UB* Lower & Upper Bounds, *β*:Standardized Estimates, *SE* Standard Error, *A* Activities

Model fit values. CMIN/DF: 8.10, RMSEA: 0.03, SRMR: 0.03, GFI: 0.95, AGFI: 0.94, NFI: 0.94, TLI: 0.94, CFI: 0.95

## Discussion

This study aimed to investigate the associative role of social development activities and the mediating role of general well-being for problematic smartphone use in the light of flourishing theory. In the structural equation model, in which perceived social support was included as a covariate, a three-step hierarchical multiple regression analysis was conducted to better understand the relationships among independent variables. The three research questions prepared for the three steps will be discussed under three headings.

### RQ 1: Is PSDA associated with PSU?

Studies have found that personal development-focused social development activities were associated with lower levels of problematic smartphone use [62, 104–107]. However, other studies have shown that entertainment and communication-focused activities were associated with higher levels of PSU [106, 108, 109]. Our findings, in line with the literature, had shown that personal development-focused activities such as physical, educational, and religious activities demonstrate a negative association with PSU, while entertainment and communication-focused activities such as social, cultural, and e-sports activities had a positive association.

Although there was no similar study in the literature, our findings also showed that voluntary activities as a personal development-focused activity did not have a significant negative association with PSU. Considering that the level of participation of participants in voluntary activities was below average, we believe that further research is needed in this regard.

When the strengths of associations were examined, it was found that the activities most strongly associated with lower levels of PSU were physical, religious, and educational activities, respectively. Therefore, it can be said that encouraging adolescents for these activities within the scope of preventive interventions may be beneficial.

Following this, it is important to consider the broader literature on the positive outcomes associated with these activities.

When examining the relationship between PSDA (Participation in Social Development Activities) and PSU (Problematic Smartphone Use) in a cross-cultural context, the literature presents various perspectives, highlighting the potential for differences across countries and cultures. Studies primarily emphasize the role of physical activities in reducing PSU [110–113], though some findings indicate complexities in this association. For a more comprehensive background, see the study by Pirwani and Szabo (2024) [114].

Smartphones today have become an integrated aspect of human life. As discussed in the literature section, smartphones can be misused, leading to problematic usage. Educational activities are regarded activities that contribute to personal development and can make life more purposeful. Although most studies support this perspective, there are contrasting findings from different countries [115, 116].

Additionally, participation in religious activities, as a variable that promotes a purposeful life, could serve as an effective approach to addressing PSU. Participation in religious activities may help individuals reflect on themselves by providing spiritual meaning or awareness. The literature offers findings from different countries that further expand our understanding [117–119].

On the other hand, the nature of social interactions has evolved in today's digital world. While past societal experiences restricted the socialization process to face-to-face interactions, today, social engagement has increasingly shifted to digital platforms. Smartphones, in particular, have played a pivotal role in reducing physical distances and diversifying social interactions through various applications. As a result, social activities have become a primary driver of increased smartphone use. Specifically, the desire to engage on social media via smartphones motivates greater participation in social activities. Our findings support this trend, though cross-cultural insights reveal variability in outcomes, as reflected in the literature. [8, 120].

Cultural tendencies vary across societies, as norms are shaped by the underlying social and historical dynamics of each community. This suggests that the relationship between cultural activities and PSU may be influenced by broader societal factors. Participation in cultural activities could potentially contribute to problematic smartphone use. One possible explanation is that smartphone technology has transcended traditional communication, evolving into an interaction-driven medium. Although the findings of the present study align with this view, cross-cultural research has yielded differing results [121].

E-sports, as an outcome of the technology age, has introduced a new perspective on the phenomenon of addiction. The purposeful use of technology can be observed in e-sports. Smartphones are readily available, making them an convenient tool for e-sports. Indeed, our findings indicated a positive relationship between e-sports and problematic smartphone use. Although research in this area remains limited, sociocultural contexts reveal variations in findings [122, 123].

In the current study, the role of voluntary activities alone was not found to be significant for PSU in a relational context. Although this was not the expectation of the research, similar studies in cross-cultural contexts have reported different results. Participation in voluntary activities is generally expected to yield positive outcomes; however, its effects may not be universal across societies. Specifically, membership in voluntary or religious organizations has been associated with strengthened feelings of security and support, playing a role in reducing digital addiction [124–128]. Thus, cross-cultural perspectives provide valuable insights into this relationship.

When direct associations were examined, it was found that while family support exhibited an indirect association with a decrease in the relationship between social activities and PSU, friend social support demonstrated an indirect association with an increase in this relationship.

Moreover, while family support was included in the model, the relationship between physical activities and PSU appeared weaker. However, since the analysis did not explicitly test moderation effects, it is more appropriate to describe this as an observed association rather than a confirmed weakening effect. Considering that physical activities associated with sports showed the weakest negative association with PSU, while e-sports activities demonstrated the strongest positive association, we argue that labeling e-sports activities as "sports" is misleading and misrepresents them as personal development- focused healthy activities. Unlike physical sports, which often involve physical exertion, teamwork, and direct social interactions, e-sports activities are primarily screen-based, sedentary, and contribute to prolonged screen time, potentially exacerbating PSU [129–132]. Studies have shown that e-sports activities are linked to increased sedentary behavior, social isolation, and heightened risk of addictive tendencies, distinguishing them from traditional sports [133–136].

Given these characteristics, the term ‘e-gaming’ rather than ‘e-sport’ is more appropriate, as it accurately reflects the nature of the activity without implying the physical or social benefits typically associated with sports [137, 138]. However, considering that 'e-gaming' is a broad term encompassing various digital gaming activities, while e-sports specifically refers to competitive gaming with monetary incentives and professional structures, we propose introducing a new term, 'professional e-gaming,' to better define this activity [139]. This term highlights the unique aspects of e-sports, distinguishing it from casual gaming by emphasizing financial incentives, organized competitions, and professional engagement [140].

Moreover, flourishing theory emphasizes the importance of meaningful engagement, social connection, and physical vitality in promoting well-being [54]. In contrast, e-sports activities may not align with these principles, as they often lack participation and holistic development elements found in traditional sports [141]. Reclassifying these activities as ‘professional e-gaming’ not only prevents misrepresentation but also underscores the need for targeted interventions addressing their unique impact on PSU.

### RQ 2: Is GWB a mediator in the relationship between PSDA and PSU?

Previous studies have shown a positive relationship between GWB and other variables [142–144] and a negative relationship with PSU [30, 81]. However, although there are some related studies [44, 145], we have not come across a study in the literature directly showing that GWB mediates between PSDA and PSU. Our findings indicate that general well-being plays a negative mediating role in the relationships between PSDA and PSU. Therefore, it can be said that PSDA associated with lower levels of PSU to the extent that it is linked to increases in GWB. The flourishing theory suggests that focusing on increasing individuals' general well-being may represent a sustainable way to address and improve mental health disorders [146, 147]. It points out that interventions focused on curing disorders often do not yield good results and may even lead to an increase in disorders, especially those associated with addiction, due to their labeling aspect [148, 149]. We see that our study's findings are aligned with this theory.

### RQ 3: Is PSS a covariate in the relationship between PSDA and PSU?

There are some studies indicating that perceived social support (PSS) is positively associated with PSDA [150] and GWB [45, 151], while it is negatively associated with PSU [152, 153]. However, studies examining the sub-dimensions of PSS separately are quite limited [96]. We did not come across a study in which PSS was examined as a covariate variable, as in our study model. Our findings indicate that family support demonstrates both direct and indirect associations with lower levels of PSU. Additionally, it has been observed that the positive association of friend social support with PSU was found to be indirectly related to a negative association through GWB. PSS plays a significant role in some PSDA's associations with lower levels of PSU.

When direct associations were examined, it was found that while family support shows an indirect association with a decrease in the relationship between social activities and PSU, while friend social support shows an indirect association with an increase., friend social support is associated with an increase. Interestingly, family support weakens the negative association of physical activities with PSU. When both family and friend support were included in the model, the positive association of social activities with PSU was lessened, while the negative association of physical activities became more pronounced. The insignificant negative association of religious activities is noteworthy.

When indirect negative associations were examined, it was observed that including only family support in the model resulted in weakening of the negative associations of PSDA with PSU in all cases. This indicates that family support is an important component of general well-being and supports the flourishing theory [54]. Because family support is associated with increases in GWB, it has a role in the relationships between PSDA and PSU. Conversely, including only friend support in the model resulted in a weakening of the negative association of social activities with PSU within PSDA. Therefore, friend social support shows an indirect association with the negative relationship between social activities and PSU through GWB, aligning the flourishing theory once again [54]. When both family and friend social support were included in the model, a weakening of the associations of physical, educational, and religious activities with PSU was observed. Both family and friend social support show indirect associations with the negative relationships between these activities and PSU through GWB. Therefore, it can be said that the flourishing theory is supported.

The potential impact of the current study outputs on policy and practice should be productively evaluated. Policymakers and public health officials can develop targeted and evidence-based methods to combine personal development-oriented activities, such as physical, educational and religious activities, with comprehensive intervention approaches targeting a preventive approach to problematic smartphone use (PSU) [154]. Social development-oriented activities should not only foster individual well-being, but also support active and direct engagement in real-world, offline interactions to counteract the isolation effects associated with problematic smartphone use. [155].

For example, governments and sample-related institutions could collaborate to produce structured programs that incorporate regular physical exercise, community-oriented educational workshops, and religious or spiritual activities appropriate for different age groups [156]. Schools and, beyond that, supportive youth centres could offer extracurricular activities that emphasize individual development, while community organizations could focus on developing volunteer and cultural activities that strengthen social belonging and a sense of purpose in life [157]. By encouraging active participation in such activities, these strategies can act as a buffer in reducing the risk of problematic smartphone use for young people who are more prone to digital technology use [158].

Further research is clearly needed to explore the cultural mechanisms that may drive the effectiveness of social development activities that offer the potential to be a preventive intervention for problematic smartphone use [159]. Cross-cultural research is critical to understand how sociological norms, values and expectations shape the effectiveness of these intervention strategies [160]. For example, in some cultures, engagement in religious activities may be more critical in promoting well-being and reducing problematic smartphone use, while in others physical or educational activities may be more beneficial. Such cross-cultural contextualization can help build cultural perspectives and best practices for micro-inferences in intervention strategies.

Furthermore, long-term and comparative studies can provide a comprehensive look at how differences in socioeconomic status, access to resources and technological sophistication of societies affect participation in social development activities and their impact on PSU [161]. Understanding these variables will help policymakers develop more inclusive and effective strategies. Finally, the findings of this study also point to the importance of interdisciplinary collaborations between educators, local authorities, mental health professionals and, furthermore, technology developers in the process of developing multidimensional strategies to address PSU.

## Conclusion

The problem of problematic smartphone use is rapidly increasing, yet it is evident that standardized interventions, rooted in the medical model and relying on uniform, treatment-focused approaches, often fall short in providing effective, sustainable, and accessible solutions – much like other addictions and associated disorders [162]. The medical model, which primarily focuses on treating symptoms through standardized interventions, often fails to consider the individual’s broader social and developmental contexts. This can lead to interventions that lack long-term effectiveness and inadvertently reinforce issues through labeling [162]. In contrast, the flourishing theory emphasizes enhancing general well-being as a sustainable approach, which is supported by the findings of this study. Further research is needed to better understand how to enhance the longevity, effectiveness, and pervasiveness of proposed intervention methods and to develop new approaches. However, above all, we believe that the persistence of the medical model in the approaches of service providers may act as a barrier to advancing these efforts. It is clear that coping with the breathtaking pace of digitalization in the past two decades requires approaches that go beyond traditional models from a century ago. Therefore, adopting new approaches could be associated with improvements in the effectiveness of intervention methods and reductions in resource waste.

In this study, we grounded our approach on the flourishing theory, and examined how relationships may vary across contexts. Our findings suggest that general well-being may play a mediating role in the relationship between participation in social development activities and problematic smartphone use. Similarly, general well-being may also mediate the relationship between social support and problematic smartphone use. General wellbeing is individual and should be addressed as such. For instance, participation in physical activities may support the general wellbeing of some individuals, while it might not have the same association for others. Therefore, it would not be reasonable to mandate participation in physical activities for everyone. Moreover, the perception of well-being is shaped by deeply personal and subjective factors, such as cultural background, personal experiences, and unique emotional landscapes [163]. Factors shaping general well-being, such as the meaning of life, life purpose, points of pleasure, criteria for appreciation, rationality, and emotionality, differ for each individual. This individuality underscores the necessity for flexible, person-centered approaches in intervention strategies, as a one-size-fits-all model risks alienating those whose needs do not align with standardized methods [164]. Thus, addressing problematic smartphone use and supporting individuals who engage in problematic smartphone behaviors requires responding to individual needs and aiming to enhance the individual's general wellbeing. As emphasized by the flourishing theory, when intervention aims to solve the problem with medical methods rather than supporting the individual's development, interventions may lack effectiveness and risk complicating the process by labeling individuals. Interventions rooted in the flourishing theory emphasize the enhancement of well-being through tailored, growth-oriented strategies, which are more likely to yield sustainable outcomes by resonating with individuals' intrinsic values and life contexts [146].

## Assumptions, Limitations, and Suggestions for Future Studies

It is assumed that participants understood all questions in the study and responded honestly. The number of participants provides a basis for the study. The literature on the relationship between social development activities and problematic smartphone use is relatively limited. We believe this limitation may result from the lack of valid and reliable scales. Developing new scales in this regard and testing them in longitudinal studies could contribute to a better understanding of and enhancing efforts aimed at addressing problematic smartphone use.

## Data Availability

The data has been anonymized, and if necessary, it can be requested from the corresponding author.

## Declarations

### Confict of Interest

The authors have no relevant fnancial or non-fnancial interests to disclose.

## References

[1] Reid AJ. The Smartphone Paradox. Springer International Publishing; 2018. 10.1007/978-3-319-94319-0

[2] O’dea S. Number of Smartphone Subscriptions Worldwide from 2016 to 2027. Statista. 2022. https://www.statista.com/statistics/330695/number-of-smartphone-users-worldwide/

[3] O’dea S. Number of smartphone users by leading countries in 2021 (in millions)*. Statista. 2022. https://www.statista.com/statistics/748053/worldwide-top-countries-smartphone-users/

[4] O’dea S. Penetration rate of smartphones in selected countries 2021. Statista. 2022. https://www.statista.com/statistics/539395/smartphone-penetration-worldwide-by-country/

[5] Kemp S. Digital 2022: Turkey. Datareportal. 2022. https://datareportal.com/reports/digital-2022-turkey

[6] TÜİK. Çocuklarda Bilişim Teknolojileri Kullanım Araştırması. 2021. https://data.tuik.gov.tr/Bulten/Index?p=Cocuklarda-Bilisim-Teknolojileri-Kullanim-Arastirmasi-2021-41132

[7] Billieux J, Philippot P, Schmid C, Maurage P, De Mol J, Van der Linden M. Is Dysfunctional Use of the Mobile Phone a Behavioural Addiction? Confronting Symptom-Based Versus Process-Based Approaches. Clin Psychol Psychother. 2015;22(5):460–8. 10.1002/CPP.1910. 24947201 10.1002/cpp.1910

[8] Elhai JD, Dvorak RD, Levine JC, Hall BJ. Problematic smartphone use: A conceptual overview and systematic review of relations with anxiety and depression psychopathology. J Affect Disord. 2017;207:251–9. 10.1016/J.JAD.2016.08.030. 27736736 10.1016/j.jad.2016.08.030

[9] Meng SQ, Cheng JL, Li YY, et al. Global prevalence of digital addiction in general population: A systematic review and meta-analysis. Clin Psychol Rev. 2022;92:102128. 10.1016/j.cpr.2022.102128. 35150965 10.1016/j.cpr.2022.102128

[10] Long J, Liu Y, Wang Y, et al. The Mediating Effects of Perceived Family Support in the Relationship Between Anxiety and Problematic Smartphone Use: A Cross-Cultural Validation. J Nerv Ment Dis. 2024;212(2):76–83. 10.1097/NMD.0000000000001738. 38030146 10.1097/NMD.0000000000001738

[11] Yang Z, Asbury K, Griffiths MD. Do Chinese and British University Students Use Smartphones Differently? A Cross-cultural Mixed Methods Study. Int J Ment Health Addict. 2019;17(3):644–57. 10.1007/s11469-018-0024-4.

[12] Olson JA, Sandra DA, Colucci ÉS, et al. Smartphone addiction is increasing across the world: A meta-analysis of 24 countries. Comput Hum Behav. 2022;129:107138. 10.1016/J.CHB.2021.107138.

[13] Dinç M. Teknoloji Bağımlılığı ve Biz. Türkiye Yeşilay Cemiyeti; 2017.

[14] Cielo F, Ulberg R, Di Giacomo D. Psychological Impact of the COVID-19 Outbreak on Mental Health Outcomes among Youth: A Rapid Narrative Review. Int J Env Res Public Health. 2021;18. 10.3390/ijerph18116067 10.3390/ijerph18116067 PMC8200066 34199896

[15] Ghogare A, Aloney S, Vankar G, Bele A, Patil P, Ambad R. A cross-sectional online survey of an impact of COVID-19 lockdown on smartphone addiction and nomophobia among undergraduate health sciences students of a rural tertiary health-care center from Maharashtra, India. Ann Indian Psychiatry. 2022;6(1). 10.4103/aip.aip_38_21

[16] Wickord LC, Quaiser-Pohl CM. Does the Type of Smartphone Usage Behavior Influence Problematic Smartphone Use and the Related Stress Perception? Behav Sci. 2022;12(4):99. 10.3390/bs12040099. 35447671 10.3390/bs12040099 PMC9032299

[17] Ahmed I, Qazi TF, Perji KA. Mobile phone to youngsters: Necessity or addiction. Afr J Bus Manag. 2011;5(32):12512–9. 10.5897/AJBM11.626.

[18] Billieux L, Van Der Linden M, Rochat L. The Role of Impulsivity in Actual and Problematic Use of the Mobile Phone. Appl Cogn Psychol. 2008;22:1195–210. 10.1002/acp.1429.

[19] Casey BM. Linking Psychological Attributes to Smartphone Addiction, Face-to-Face Communication. Present Absence and Social Capital: The Chinese University of Hong Kong; 2012.

[20] Hong FY, Chiu SI, Huang DH. A model of the relationship between psychological characteristics, mobile phone addiction and use of mobile phones by Taiwanese university female students. Comput Hum Behav. 2012;28(6):2152–9. 10.1016/J.CHB.2012.06.020.

[21] Krajewska-Kułak E, Kułak W, Stryzhak A, Szpakow A, Prokopowicz W, Marcinkowski J. Problematic mobile phone using among the Polish and Belarusian University students, a comparative study. Prog Health Sci. 2012;2(1):45–50.

[22] Kwon M, Lee JY, Won WY, et al. Development and Validation of a Smartphone Addiction Scale (SAS). PLoS ONE. 2013;8(2):e56936. 10.1371/JOURNAL.PONE.0056936. 23468893 10.1371/journal.pone.0056936 PMC3584150

[23] Park WK. Mobile Phone Addiction. In: Mobile Communications. Springer, London; 2005;31:253–272. 10.1007/1-84628-248-9_17

[24] Szpakow A, Stryzhak A, Prokopowicz W. Evaluation of threat of mobile phone-addition among Belarusian University students. Prog Health Sci. 2011;1(2):96–101.

[25] Takao M, Takahashi S, Kitamura M. Addictive personality and problematic mobile phone use. Cyberpsychology Behav Impact Internet Multimed Virtual Real Behav Soc. 2009;12(5):501–7. 10.1089/CPB.2009.0022. 10.1089/cpb.2009.0022 19817562

[26] Vezzoli M, Colombo A, Marano A, Zoccatelli G, Zogmaister C. Test for Mobile phone dependence: psychometric properties and confirmatory factor analysis. Curr Psychol. Published online February 2021:1–12. 10.1007/S12144-021-01449-5/TABLES/4

[27] Yu S, Sussman S. Does Smartphone Addiction Fall on a Continuum of Addictive Behaviors? Int J Environ Res Public Health. 2020;17(2):422. 10.3390/IJERPH17020422. 31936316 10.3390/ijerph17020422 PMC7014405

[28] Panova T, Carbonell X. Is smartphone addiction really an addiction? J Behav Addict. 2018;7(2):252–9. 10.1556/2006.7.2018.49. 29895183 10.1556/2006.7.2018.49 PMC6174603

[29] Nawaz S. Rethinking classifications and metrics for problematic smartphone use and dependence: Addressing the call for reassessment. Comput Hum Behav Rep. 2023;12:100327. 10.1016/j.chbr.2023.100327.

[30] Horwood S, Anglim J. Problematic smartphone usage and subjective and psychological well-being. Comput Hum Behav. 2019;97:44–50. 10.1016/J.CHB.2019.02.028.

[31] Longo Y, Coyne I, Joseph S. Development of the short version of the Scales of General Well-Being: The 14-item SGWB. Personal Individ Differ. 2018;124:31–4. 10.1016/j.paid.2017.11.042.

[32] Kalafatoğlu MR, Balcı Çelik S. Genel İyi Oluş Ölçeği Kısa Formu’nun Türkçe’ye Uyarlanması: Geçerlik ve Güvenirlik Çalışması. OPUS Uluslar Toplum Araştırmaları Derg. Published online May 31, 2020:1–1. 10.26466/opus.644835

[33] Gao T, Ding X, Chai J, et al. The influence of resilience on mental health: The role of general well-being. Int J Nurs Pract. 2017;23(3):e12535. 10.1111/ijn.12535. 10.1111/ijn.12535 28294467

[34] Xia X, Qin S, Zhang S. Leisure Experience and Mobile Phone Addiction: Evidence from Chinese Adolescents. Heliyon. 2024;10(3):e24834. 10.1016/j.heliyon.2024.e24834. 38317952 10.1016/j.heliyon.2024.e24834 PMC10839556

[35] Holahan CJ, Moos RH. Social support and adjustment: Predictive benefits of social climate indices. Am J Community Psychol. 1982;10(4):403–15. 10.1007/BF00893979. 7137128 10.1007/BF00893979

[36] Hargie O, Saunders C, Dickson D. Social Skills in Interpersonal Communication. Third. Routledge; 1994. https://books.google.com.tr/books?id=vdH8vjQvM-MC

[37] Fleming CM, Cook A. The recreational value of Lake McKenzie, Fraser Island: An application of the travel cost method. Tour Manag. 2008;29(6):1197–205. 10.1016/j.tourman.2008.02.022.

[38] Kawachi I, Berkman LF. Social ties and mental health. J Urban Health Bull N Y Acad Med. 2001;78(3):458–67. 10.1093/JURBAN/78.3.458. 10.1093/jurban/78.3.458 PMC3455910 11564849

[39] Bainbridge WS. The Religious Ecology of Deviance. Am Sociol Rev. 1989;54(2):288. 10.2307/2095796.

[40] Abjam Z, Soltani A, Dehghan H. Developing a Model for Promoting Social Responsibility of the Red Crescent Society by Emphasizing on Volunteers’ Participation in Disasters. Jorar. 2023;15(3):216–25. 10.32592/jorar.2023.15.3.7.

[41] Yip C, Sarma S, Wilk P. The Association between Social Cohesion and Physical Activity in Canada: A Multilevel Analysis. SSM - Popul Health. 2016;2:718–23. 10.1016/j.ssmph.2016.09.010. 29349183 10.1016/j.ssmph.2016.09.010 PMC5757883

[42] Fischer G. Understanding, Fostering, and Supporting Cultures of Participation. Interactions. 2011;18(3):42–53. 10.1145/1962438.1962450.

[43] Hamilton-West T. Motivational Orientations of RN-BSN Students at Rowan University. Master: Rowan University; 2013.

[44] Zhong W, Wang Y, Zhang G. The Impact of Physical Activity on College Students’ Mobile Phone Dependence: the Mediating Role of Self-Control. Int J Ment Health Addict. 2021;19(6):2144–59. 10.1007/s11469-020-00308-x.

[45] Şimşek OM, Kaya AB, Çevik O, Koçak O. How is the problematic smartphone use affected by social support? A research model supported by the mediation of Ikigai. Curr Psychol. Published online February 18, 2023. 10.1007/s12144-023-04362-1 10.1007/s12144-023-04362-1 PMC9938517 36845209

[46] Zhou Z, Cheng Q. Relationship between online social support and adolescents’ mental health: A systematic review and meta-analysis. J Adolesc. 2022;94(3):281–92. 10.1002/jad.12031. 35390193 10.1002/jad.12031

[47] Feng L, Yin R. Social Support and Hope Mediate the Relationship Between Gratitude and Depression Among Front-Line Medical Staff During the Pandemic of COVID-19. Front Psychol. 2021;12:623873. 10.3389/fpsyg.2021.623873. 33776846 10.3389/fpsyg.2021.623873 PMC7987792

[48] Grey I, Arora T, Thomas J, Saneh A, Tohme P, Abi-Habib R. The role of perceived social support on depression and sleep during the COVID-19 pandemic. Psychiatry Res. 2020;293:113452. 10.1016/j.psychres.2020.113452. 32977047 10.1016/j.psychres.2020.113452 PMC7500407

[49] Leow K, Lynch MF, Lee J. Social Support, Basic Psychological Needs, and Social Well-Being Among Older Cancer Survivors. Int J Aging Hum Dev. 2021;92(1):100–14. 10.1177/0091415019887688. 31718228 10.1177/0091415019887688

[50] Yeung NC, Chow TS. Coping with my own way: Mediating roles of emotional expression and social support seeking in the associations between individual differences and posttraumatic growth. Health Psychol Open. 2019;6(1):2055102919846596. 10.1177/2055102919846596. 31105967 10.1177/2055102919846596 PMC6503603

[51] Forster M, Grigsby TJ, Gower AL, Mehus CJ, McMorris BJ. The Role of Social Support in the Association between Childhood Adversity and Adolescent Self-injury and Suicide: Findings from a Statewide Sample of High School Students. J Youth Adolesc. 2020;49(6):1195–208. 10.1007/s10964-020-01235-9. 32297174 10.1007/s10964-020-01235-9

[52] Pössel P, Burton SM, Cauley B, Sawyer MG, Spence SH, Sheffield J. Associations between Social Support from Family, Friends, and Teachers and depressive Symptoms in Adolescents. J Youth Adolesc. 2018;47(2):398–412. 10.1007/s10964-017-0712-6. 28695369 10.1007/s10964-017-0712-6

[53] McEntee ML, Dy-Liacco GS, Haskins DG. Human Flourishing: A Natural Home for Spirituality. J Spiritual Ment Health. 2013;15(3):141–59. 10.1080/19349637.2013.799410.

[54] Keyes CLM, Haidt J, editors. Flourishing*:* Positive Psychology and the Life Well-Lived*.* American Psychological Association; 2003. 10.1037/10594-000

[55] Seligman MEP. Flourish: A Visionary New Understanding of Happiness and Well-Being. Reprint. Free Press; 2012. Accessed October 1, 2023. http://gen.lib.rus.ec/book/index.php?md5=b48ed8ac6c904fe2c646033934d19720

[56] Keyes C. Why Flourishing? In: Well-Being and Higher Education. 1st ed. Brining Theory to Practice; 2016:99–107.

[57] Lyubomirsky S, Tkach C, DiMatteo MR. What are the differences between happiness and self-esteem? Soc Indic Res. 2006;78(3):363–404. 10.1007/s11205-005-0213-y.

[58] Khaw D, Kern ML. A Cross-Cultural Comparison of the PERMA Model of Well-Being. Undergrad J Psychol Berkeley. 2014;8:10–23.

[59] Fredrickson BL. The Role of Positive Emotions in Positive Psychology. Am Psychol. 2001;56(3):218–26. 11315248 10.1037//0003-066x.56.3.218 PMC3122271

[60] Abbasi GA, Jagaveeran M, Goh YN, Tariq B. The impact of type of content use on smartphone addiction and academic performance: Physical activity as moderator. Technol Soc. 2021;64:101521. 10.1016/J.TECHSOC.2020.101521.

[61] Baskan Ö, Çorum M, Büyükyilmaz G. Üniversite Öğrencilerinde Akıllı Telefon Bağımlılığı ile Fiziksel Aktivite, Yorgunluk ve Uyku Kalitesinin İlişkisinin İncelenmesi. Gümüşhane Üniversitesi Sağlık Bilim Derg. 2023;12(1):299–305. 10.37989/gumussagbil.1049962.

[62] Gedi̇k S, Gezgi̇n DM. Üniversite Öğrencilerinin Akıllı Telefon Bağımlılığının Rekreatif Faaliyetlere Katılım Davranışları Açısından İncelenmesi. Genç Araştırmaları Derg. 2022;10(28):1–20. 10.52528/genclikarastirmalari.973348.

[63] Kim H. Exercise rehabilitation for smartphone addiction. J Exerc Rehabil. 2013;9(6):500. 10.12965/JER.130080. 24409425 10.12965/jer.130080 PMC3884868

[64] Pereira FS, Bevilacqua GG, Coimbra DR, Andrade A. Impact of Problematic Smartphone Use on Mental Health of Adolescent Students: Association with Mood, Symptoms of Depression, and Physical Activity. Cyberpsychology Behav Soc Netw. 2020;23(9):619–26. 10.1089/cyber.2019.0257. 10.1089/cyber.2019.0257 32580574

[65] Shim JY. Christian Spirituality and Smartphone Addiction in Adolescents: A Comparison of High-Risk, Potential-Risk, and Normal Control Groups. J Relig Health. 2019;58(4):1272–85. 10.1007/s10943-018-00751-0. 30610513 10.1007/s10943-018-00751-0

[66] Greco G, Tambolini R, Ambruosi P, Fischetti F. Negative effects of smartphone use on physical and technical performance of young footballers. *J Phys Educ Sport*. 17(04).

[67] Coffey JK, Wray-Lake L, Mashek D, Branand B. A Multi-Study Examination of Well-Being Theory in College and Community Samples. J Happiness Stud. 2016;17(1):187–211. 10.1007/s10902-014-9590-8.

[68] Schotanus-Dijkstra M, Pieterse ME, Drossaert CHC, et al. What Factors are Associated with Flourishing? Results from a Large Representative National Sample. J Happiness Stud. 2016;17(4):1351–70. 10.1007/s10902-015-9647-3.

[69] Keyes CLM. Social Well-Being. Soc Psychol Q. 1998;61(2):121–40. 10.2307/2787065.

[70] Momtaz YA, Hamid TA, Haron SA, Bagat MF. Flourishing in later life. Arch Gerontol Geriatr. 2016;63:85–91. 10.1016/j.archger.2015.11.001. 26627531 10.1016/j.archger.2015.11.001

[71] Bjursell C. Inclusion in education later in life: Why older adults engage in education activities. Eur J Res Educ Learn Adults. 2019;10(3):215–30. 10.3384/rela.2000-7426.rela20192.

[72] Burke M, Marlow C, Lento T. Social network activity and social well-being. In: Proceedings of the SIGCHI Conference on Human Factors in Computing Systems. CHI ’10. Association for Computing Machinery; 2010:1909–1912. 10.1145/1753326.1753613

[73] Cheng M, Chen L, Pan Q, Gao Y, Li J. E−sports playing and its relation to lifestyle behaviors and psychological well-being: A large-scale study of collegiate e-sports players in China. Complement Ther Clin Pract. 2023;51:101731. 10.1016/j.ctcp.2023.101731. 36716672 10.1016/j.ctcp.2023.101731

[74] McAuley E, Rudolph D. Physical Activity, Aging, and Psychological Well-Being. J Aging Phys Act. 1995;3(1):67–96. 10.1123/japa.3.1.67.

[75] Molsher R, Townsend M. Improving Wellbeing and Environmental Stewardship Through Volunteering in Nature. EcoHealth. 2016;13(1):151–5. 10.1007/s10393-015-1089-1. 26678275 10.1007/s10393-015-1089-1

[76] Littman-Ovadia H, Steger M. Character strengths and well-being among volunteers and employees: Toward an integrative model. J Posit Psychol. 2010;5(6):419–30. 10.1080/17439760.2010.516765.

[77] Poloma MM, Pendleton BF. Religious domains and general well-being. Soc Indic Res. 1990;22(3):255–76. 10.1007/BF00301101.

[78] Trainor S, Delfabbro P, Anderson S, Winefield A. Leisure activities and adolescent psychological well-being. J Adolesc. 2010;33(1):173–86. 10.1016/j.adolescence.2009.03.013. 19406463 10.1016/j.adolescence.2009.03.013

[79] Wheatley D, Bickerton C. Subjective well-being and engagement in arts, culture and sport. J Cult Econ. 2017;41(1):23–45. 10.1007/s10824-016-9270-0.

[80] Kumcağiz AH, Gündüz AY. Relationship between Psychological Well-Being and Smartphone Addiction of University Students. Int J High Educ. 2016;5(4):144–56. 10.5430/ijhe.v5n4p144.

[81] Mascia ML, Agus M, Penna MP. Emotional Intelligence, Self-Regulation, Smartphone Addiction: Which Relationship With Student Well-Being and Quality of Life? Front Psychol. 2020;11:375. 10.3389/fpsyg.2020.00375. 32210888 10.3389/fpsyg.2020.00375 PMC7068808

[82] Chu PS, Saucier DA, Hafner E. Meta-Analysis of the Relationships Between Social Support and Well-Being in Children and Adolescents. J Soc Clin Psychol. 2010;29(6):624–45. 10.1521/JSCP.2010.29.6.624.

[83] Heerde JA, Hemphill SA. Examination of associations between informal help-seeking behavior, social support, and adolescent psychosocial outcomes: A meta-analysis. Dev Rev. 2018;47:44–62. 10.1016/j.dr.2017.10.001.

[84] Effects of peer victimization in schools and perceived social support on adolescent well‐being - RIGBY - 2000 - Journal of Adolescence - Wiley Online Library. October 9, 2023. Accessed October 9, 2023. https://onlinelibrary.wiley.com/doi/abs/10.1006/jado.1999.0289 10.1006/jado.1999.0289 10700372

[85] van Wel F, Linssen H, Abma R. The Parental Bond and the Well-Being of Adolescents and Young Adults. J Youth Adolesc. 2000;29(3):307–18. 10.1023/A:1005195624757.

[86] Yarcheski A, Mahon NE, Yarcheski TJ. Stress, Hope, and Loneliness in Young Adolescents: *Httpdxorg102466020709PR01083919–922*. 2011;108(3):919–922. 10.2466/02.07.09.PR0.108.3.919-922 10.2466/02.07.09.PR0.108.3.919-922 21879638

[87] Riahi ME, Aliverdinia A, Pourhossein Z. Relationship between Social Support and Mental Health. Soc Welf Q. 2011;10(39):85–121.

[88] Al-Kandari YY, Al-Sejari MM. Social isolation, social support and their relationship with smartphone addiction. Inf Commun Soc. 2020;24(13):1925–43. 10.1080/1369118X.2020.1749698.

[89] Herrero J, Torres A, Vivas P, Urueña A. Smartphone Addiction and Social Support: A Three-year Longitudinal Study. Psychosoc Interv. 2019;28(3):111–8. 10.5093/PI2019A6. 10.5093/pi2022a3 PMC10268539 37362618

[90] Kelly ME, Duff H, Kelly S, et al. The impact of social activities, social networks, social support and social relationships on the cognitive functioning of healthy older adults: a systematic review. Syst Rev. 2017;6(1):259. 10.1186/s13643-017-0632-2. 29258596 10.1186/s13643-017-0632-2 PMC5735742

[91] Berkman LF, Glass T, Brissette I, Seeman TE. From social integration to health: Durkheim in the new millennium. Soc Sci Med. 2000;51(6):843–57. 10.1016/S0277-9536(00)00065-4. 10972429 10.1016/s0277-9536(00)00065-4

[92] Leavy P. Research Design. THE GUILFORD PRESS; 2017.

[93] Sample size calculator. Raosoft. 2004. http://www.raosoft.com/samplesize.html

[94] Csibi S, Griffiths MD, Cook B, Demetrovics Z, Szabo A. The Psychometric Properties of the Smartphone Application-Based Addiction Scale (SABAS). Int J Ment Health Addict. 2018;16(2):393–403. 10.1007/S11469-017-9787-2/TABLES/5. 29670500 10.1007/s11469-017-9787-2 PMC5897481

[95] Gökler ME, Bulut YE. Akıllı Telefon Uygulamasına Dayalı Bağımlılık Ölçeği’nin Türkçe Sürümünün Geçerlilik ve Güvenilirliğinin Değerlendirilmesi. JCBPR. 2019;8(2):100–6. 10.5455/JCBPR.38288.

[96] Zimet GD, Dahlem NW, Zimet SG, Farley GK. The Multidimensional Scale of Perceived Social Support. J Pers Assess. 1988;52(1):30–41. 10.1207/S15327752JPA5201_2.

[97] Eker D, ve Yaldız Arkar H, H,. Çok Boyutlu Algılanan Sosyal Destek Ölçeği’nin Gözden Geçirilmiş Formunun Faktör Yapısı. Geçerlik ve Güvenirliği Türk Psikiyatri Derg. 2001;12(1):17–25.

[98] Ghaleb M, Yaslioglu M. Structural Equation Modeling (SEM) for Social and Behavioral Sciences Studies: Steps Sequence and Explanation. *J Organ Behav Rev*. 2024;6(1):69–108. https://dergipark.org.tr/en/pub/jobreview/issue/82893/1395927. Accessed 31 Dec 2023

[99] Hair JF, Black WC, Babin BJ, Anderson RE. Multivariate Data Analysis. Pearson. 2014. 10.1016/j.ijpharm.2011.02.019.

[100] Weston R, Gore PA. A Brief Guide to Structural Equation Modeling. Couns Psychol. 2006;34(5):719–51. 10.1177/0011000006286345.

[101] Leyland AH, Groenewegen PP. *Multilevel Modelling for Public Health and Health Services Research: Health in Context*. Springer International Publishing; 2020. https://books.google.com.tr/books?id=o5HTDwAAQBAJ 33347097

[102] Tisak J. Determination of the Regression Coefficients and Their Associated Standard Errors in Hierarchical Regression Analysis. Multivar Behav Res. 1994;29(2):185–201. 10.1207/s15327906mbr2902_4. 10.1207/s15327906mbr2902_4 26745027

[103] Kline RB. *Principles and Practice of Structural Equation Modeling*. Guilford Press; 2011.

[104] Dossi F, Buja A, Montecchio L. Association between religiosity or spirituality and internet addiction: A systematic review. Front Public Health. 2022;10:980334. 10.3389/fpubh.2022.980334. 36530734 10.3389/fpubh.2022.980334 PMC9751319

[105] Gabor AM, Mikloušić I. Using Technology to Predict Leisure Activities and Quality of Life. In: Wac K, Wulfovich S, editors. Quantifying Quality of Life. Health Informatics. Springer International Publishing; 2022:511–522. 10.1007/978-3-030-94212-0_22

[106] Meng H, Cao H, Hao R, et al. Smartphone use motivation and problematic smartphone use in a national representative sample of Chinese adolescents: The mediating roles of smartphone use time for various activities. J Behav Addict. 2020;9(1):163–74. 10.1556/2006.2020.00004. 32359238 10.1556/2006.2020.00004 PMC8935195

[107] Precht LM, Mertens F, Brickau DS, et al. Engaging in physical activity instead of (over)using the smartphone: An experimental investigation of lifestyle interventions to prevent problematic smartphone use and to promote mental health. *J Public Health*. Published online February 9, 2023. 10.1007/s10389-023-01832-5 10.1007/s10389-023-01832-5 PMC9909154 36785655

[108] Can HC, Tekkurşun Demi̇r G. Sporcuların ve E-spor Oyuncularının Dijital Oyun Bağımlılığı ve Dijital Oyun Bağımlılığına İlişkin Farkındalık Düzeyleri. Gaziantep Üniversitesi Spor Bilim Derg. 2020;5(4):364-384. 10.31680/gaunjss.770600

[109] Maldonado-Murciano L, Guilera G, Montag C, Pontes HM. Disordered gaming in esports: Comparing professional and non-professional gamers. Addict Behav. 2022;132:107342. 10.1016/j.addbeh.2022.107342. 35584554 10.1016/j.addbeh.2022.107342

[110] Ali SZ, Ali A. Relationship between Physical Activity and WhatsApp Addiction among Athletes and Non Athletes of University Level Early Adults. J Dev Soc Sci. 2023;4(3). 10.47205/jdss.2023(4-III)43

[111] Buke M, Egesoy H, Unver F. The effect of smartphone addiction on physical activity level in sports science undergraduates. J Bodyw Mov Ther. 2021;28:530–4. 10.1016/j.jbmt.2021.09.003. 34776190 10.1016/j.jbmt.2021.09.003

[112] Guo KL, Ma QS, Yao SJ, et al. The Relationship Between Physical Exercise and Mobile Phone Addiction Tendency of University Students in China: A Moderated Mediation Model. Front Psychol. 2022;13:730886. 10.3389/fpsyg.2022.730886. 35237204 10.3389/fpsyg.2022.730886 PMC8884265

[113] Lepp A, Barkley JE, Sanders GJ, Rebold M, Gates P. The relationship between cell phone use, physical and sedentary activity, and cardiorespiratory fitness in a sample of U.S. college students. Int J Behav Nutr Phys Act. 2013;10(1):79. 10.1186/1479-5868-10-79. 23800133 10.1186/1479-5868-10-79 PMC3693866

[114] Pirwani N, Szabo A. Could physical activity alleviate smartphone addiction in university students? A systematic literature review. Prev Med Rep. 2024;42:102744. 10.1016/j.pmedr.2024.102744. 38707250 10.1016/j.pmedr.2024.102744 PMC11068924

[115] Ertemel AV, Ari E. A Marketing Approach to a Psychological Problem: Problematic Smartphone Use on Adolescents. Int J Environ Res Public Health. 2020;17(7):2471. 10.3390/ijerph17072471. 32260429 10.3390/ijerph17072471 PMC7177352

[116] Kent S, Masterson C, Ali R, Parsons CE, Bewick BM. Digital Intervention for Problematic Smartphone Use. Int J Environ Res Public Health. 2021;18(24):13165. 10.3390/ijerph182413165. 34948774 10.3390/ijerph182413165 PMC8701454

[117] Pratiwi NE, Heriyudanta M, Daryono RW. The Impact of Islamic Religious Learning and Social Media Distraction on Procrastination Behavior in Higher Education : Does the Screen Time Mediation Matter? J Paedagogy. 2024;11(3):496. 10.33394/jp.v11i3.11284.

[118] Safaria T, Saputra NE, Arini DP. The Impact of Nomophobia: Exploring the Interplay Between Loneliness, Smartphone Usage, Self-control, Emotion Regulation, and Spiritual Meaningfulness in an Indonesian Context. J Technol Behav Sci. Published online September 11, 2024. 10.1007/s41347-024-00438-2

[119] Wijaya HE, Putri SAA, Firdausi Z, Nabila NN. Pengaruh Religiusitas Terhadap Penggunaan Gawai yang Bermasalah: Peran Kontrol Diri dan Stres Pada Mahasiswa. Psychosophia J Psychol Relig Humanity. 2021;3(2):95–111. 10.32923/psc.v3i2.1933.

[120] Kim JH. Psychological issues and problematic use of smartphone: ADHD’s moderating role in the associations among loneliness, need for social assurance, need for immediate connection, and problematic use of smartphone. Comput Hum Behav. 2018;80:390–8. 10.1016/j.chb.2017.11.025.

[121] Hughes K, Moscardo G. Connecting with New Audiences: Exploring the Impact of Mobile Communication Devices on the Experiences of Young Adults in Museums. Visit Stud. 2017;20(1):33–55. 10.1080/10645578.2017.1297128.

[122] Paik SH, Kim DJ. Smart Healthcare Systems and Precision Medicine. In: Kim YK, editors. Frontiers in Psychiatry. Vol 1192. Advances in Experimental Medicine and Biology. Springer Singapore; 2019:263–279. 10.1007/978-981-32-9721-0_13 10.1007/978-981-32-9721-0_13 31705499

[123] Young KS. The evolution of Internet addiction. Addict Behav. 2017;64:229–30. 10.1016/j.addbeh.2015.05.016. 26059165 10.1016/j.addbeh.2015.05.016

[124] Ihm J, Hsieh YP. The implications of information and communication technology use for the social well-being of older adults. Inf Commun Soc. 2015;18(10):1123–38. 10.1080/1369118X.2015.1019912.

[125] Lin N, Dean A, Ensel WM. Social Support, Life Events, and Depression. Academic Press; 1986. https://books.google.com.tr/books?id=XF49LCsJatsC

[126] Putnam RD. Bowling Alone: The Collapse and Revival of American Community. Simon & Schuster; 2000. https://books.google.com.tr/books?id=rd2ibodep7UC

[127] Samaha M, Hawi NS. Relationships among smartphone addiction, stress, academic performance, and satisfaction with life. Comput Hum Behav. 2016;57:321–5. 10.1016/J.CHB.2015.12.045.

[128] Tsai HF, Cheng SH, Yeh TL, et al. The risk factors of Internet addiction—A survey of university freshmen. Psychiatry Res. 2009;167(3):294–9. 10.1016/j.psychres.2008.01.015. 19395052 10.1016/j.psychres.2008.01.015

[129] Ketelhut S, Martin-Niedecken AL, Zimmermann P, Nigg CR. Physical Activity and Health Promotion in Esports and Gaming-Discussing Unique Opportunities for an Unprecedented Cultural Phenomenon. Front Sports Act Living. 2021;3: 693700. 10.3389/fspor.2021.693700. 34604743 10.3389/fspor.2021.693700 PMC8481377

[130] Berga D, Pereda A, De Filippi E, et al. Measuring arousal and stress physiology on Esports, a League of Legends case study. Published online. 2023. 10.48550/ARXIV.2302.14269.

[131] Can S. Sedentary Behavior, Number of Steps and Health. Turk J Sports Med. 2019;54(1):71–82. 10.5152/tjsm.2019.118.

[132] Wang S, Chiu DKW, Ho KKW. Exploring Esports game addiction among college students in multiplayer online battle arena: a quantitative model centered on self-control and motivation. Aslib J Inf Manag. Published online December 24, 2024. 10.1108/AJIM-05-2024-0392

[133] Xenopoulos P, Silva C. ESTA: An Esports Trajectory and Action Dataset. Published online. 2022. 10.48550/ARXIV.2209.09861.

[134] Ayhan B. Türkiye’de E-Spor Üzerine Yapılan Akademik Çalışmaların İçerik Analizi. *Uluslar Güncel Eğitim Araştırmaları Derg*. 2021;7(2):656–671. https://dergipark.org.tr/tr/pub/intjces/issue/67938/1036316

[135] Mustafaoğlu R. e-Spor, Spor ve Fiziksel Aktivite. Ulus Spor Bilim Derg. 2018;2(2):84–96. 10.30769/usbd.457545.

[136] Belli̇ E, Saraçoğlu Y. Espor Başlıklı Bilimsel Araştırmaların Bibliyometrik Analizi. Sportive. 2023;6(2):52–66. 10.53025/sportive.1308802

[137] Riatti P, Thiel A. The societal impact of electronic sport: a scoping review. Ger J Exerc Sport Res. 2022;52(3):433–46. 10.1007/s12662-021-00784-w. 40477796 10.1007/s12662-021-00784-w PMC8622113

[138] Marelić M, Vukušić D. E-sports: Definition and social implications. Exerc Qual Life. 2019;11(2):47–54. 10.31382/eqol.191206.

[139] Bányai F, Zsila Á, Griffiths MD, Demetrovics Z, Király O. Career as a Professional Gamer: Gaming Motives as Predictors of Career Plans to Become a Professional Esport Player. Front Psychol. 2020;11:1866. 10.3389/fpsyg.2020.01866. 32903792 10.3389/fpsyg.2020.01866 PMC7438909

[140] Delello JA, McWhorter RR, Roberts P, Dockery HS, De Giuseppe T, Corona F. The Rise of eSports: Insights Into the Perceived Benefits and Risks for College Students. Int J ESports Res. 2021;1(1):1–19. 10.4018/IJER.20210101.oa5.

[141] Pu H, Kim J, Daprano C. Can Esports Substitute Traditional Sports? The Convergence of Sports and Video Gaming during the Pandemic and Beyond. Societies. 2021;11(4):129. 10.3390/soc11040129.

[142] Jackson SB, Stevenson KT, Larson LR, Peterson MN, Seekamp E. Outdoor Activity Participation Improves Adolescents’ Mental Health and Well-Being during the COVID-19 Pandemic. Int J Environ Res Public Health. 2021;18(5):2506. 10.3390/ijerph18052506. 33802521 10.3390/ijerph18052506 PMC7967628

[143] Puchalska-Wasyl MM, Zarzycka B. Internal Dialogue as a Mediator of the Relationship Between Prayer and Well-Being. J Relig Health. 2020;59(4):2045–63. 10.1007/s10943-019-00943-2. 31707518 10.1007/s10943-019-00943-2 PMC7359138

[144] Roberts A, Hinds J, Camic PM. Nature activities and wellbeing in children and young people: a systematic literature review. J Adventure Educ Outdoor Learn. 2020;20(4):298–318. 10.1080/14729679.2019.1660195.

[145] Chen H, Wang C, Lu T, Tao B, Gao Y, Yan J. The Relationship between Physical Activity and College Students’ Mobile Phone Addiction: The Chain-Based Mediating Role of Psychological Capital and Social Adaptation. Int J Environ Res Public Health. 2022;19(15):9286. 10.3390/ijerph19159286. 35954644 10.3390/ijerph19159286 PMC9367822

[146] Keyes CLM. Promoting and protecting mental health as flourishing: A complementary strategy for improving national mental health. Am Psychol. 2007;62(2):95–108. 10.1037/0003-066X.62.2.95. 17324035 10.1037/0003-066X.62.2.95

[147] Seligman MEP. Flourish: A Visionary New Understanding of Happiness and Well-Being*.* Free Press; 2011:xii, 349.

[148] Corrigan PW, Rao D. On the Self-Stigma of Mental Illness: Stages, Disclosure, and Strategies for Change. Can J Psychiatry. 2012;57(8):464–9. 10.1177/070674371205700804. 22854028 10.1177/070674371205700804 PMC3610943

[149] Ryff CD, Singer B. The Contours of Positive Human Health. Psychol Inq. 1998;9(1):1–28. 10.1207/s15327965pli0901_1.

[150] Han A, Kim J, Kim J. Coping Strategies, Social Support, Leisure Activities, and Physical Disabilities. Am J Health Behav. 2019;43(5):937–49. 10.5993/AJHB.43.5.6. 31439100 10.5993/AJHB.43.5.6

[151] Wilson JM, Weiss A, Shook NJ. Mindfulness, self-compassion, and savoring: Factors that explain the relation between perceived social support and well-being. Personal Individ Differ. 2020;152:109568. 10.1016/j.paid.2019.109568.

[152] Aker S, Şahin MK, Sezgin S, Oğuz G. Psychosocial Factors Affecting Smartphone Addiction in University Students. J Addict Nurs. 2017;28(4):215–9. 10.1097/JAN.0000000000000197. 29200049 10.1097/JAN.0000000000000197

[153] Jennifer I. Social implications of children’s smartphone addiction: The role of support networks and social engagement. J Behav Addict. 2018;7(2):473–81. 10.1556/2006.7.2018.48. 29865865 10.1556/2006.7.2018.48 PMC6174576

[154] Twenge JM, Campbell WK. Associations between screen time and lower psychological well-being among children and adolescents: Evidence from a population-based study. Prev Med Rep. 2018;12:271–83. 10.1016/j.pmedr.2018.10.003. 30406005 10.1016/j.pmedr.2018.10.003 PMC6214874

[155] Kim JH. Smartphone-mediated communication vs. face-to-face interaction: Two routes to social support and problematic use of smartphone. Comput Hum Behav. 2017;67:282–91. 10.1016/J.CHB.2016.11.004.

[156] Çevik O, Çoltu İ, Şimşek OM. The Role of Local Governments in Struggling with Technology Addiction: Bayamer Sample. In: BZT Academy; 2023.

[157] Shamah D. Supporting a strong sense of purpose: Lessons from a rural community. New Dir Youth Dev. 2011;2011(132):45–58. 10.1002/yd.427. 22275278 10.1002/yd.427

[158] Efrati Y, Rosenberg H, Ophir Y. Effective parental strategies against problematic smartphone use among adolescents: A 6-month prospective study. Addict Behav. 2024;154:108024. 10.1016/j.addbeh.2024.108024. 38555777 10.1016/j.addbeh.2024.108024

[159] Moqbel M, Bartelt V, Alam M, Shaik AY, Montoya S, Larson M. Smartphone Addiction and Cultural Dimensions. In; 2023. 10.24251/HICSS.2023.261

[160] Harkness S, Keefer CH. Contributions of Cross-Cultural Psychology to Research and Interventions in Education and Health. J Cross-Cult Psychol. 2000;31(1):92–109. 10.1177/0022022100031001008.

[161] Zara AR. From the Digital Divide to Public Participation: Access, Use, Literacy, and Perceived Benefit in Social and Political Participation. SSRN Electron J Published online. 2024. 10.2139/ssrn.4674296.

[162] Engel GL. The Need for a New Medical Model: A Challenge for Biomedicine. Science. 1977;196(4286):129–36. 10.1126/science.847460. 847460 10.1126/science.847460

[163] Diener E, Suh EM, Lucas RE, Smith HL. Subjective well-being: Three decades of progress. Psychol Bull. 1999;125(2):276–302. 10.1037/0033-2909.125.2.276.

[164] Ryff CD, Singer BH. Know Thyself and Become What You Are: A Eudaimonic Approach to Psychological Well-Being. J Happiness Stud. 2008;9(1):13–39. 10.1007/s10902-006-9019-0.

